# Development of a Culturally Anchored Qualitative Approach to Conduct and Analyze Focus Group Narratives Collected in Diné (Navajo) Communities to Understand the Impacts of the Gold King Mine Spill of 2015

**DOI:** 10.3390/ijerph18179402

**Published:** 2021-09-06

**Authors:** Nicolette I. Teufel-Shone, Carmenlita Chief, Jennifer R. Richards, Rebecca J. Clausen, Alfred Yazzie, Manley A. Begay, Nathan Lothrop, Janene Yazzie, Andria B. Begay, Paloma I. Beamer, Karletta Chief

**Affiliations:** 1 Center for Health Equity Research, Northern Arizona University, Flagstaff, AZ 86011, USA; carmenlita.chief@nau.edu (C.C.); andria.begay@nau.edu (A.B.B.); 2 Center for American Indian Health, John Hopkins University, Baltimore, MD 21231, USA; jricha81@jhu.edu; 3 Department of Sociology and Human Services, Fort Lewis College, Durango, CO 81301, USA; clausen_r@fortlewis.edu; 4 Independent Researcher, Winslow, AZ 86047, USA; alfred.yazzie54@gmail.com; 5 Applied Indigenous Studies Department, Northern Arizona University, Flagstaff, AZ 86011, USA; Manley.Begay-Jr@nau.edu; 6 Asthma and Airway Disease Research Center and Mel and Enid Zuckerman College of Public Health, University of Arizona, Tucson, AZ 85724, USA; lothrop@arizona.edu (N.L.); pbeamer@arizona.edu (P.I.B.); 7 Tó Bee Nihi Dziil and Sixth World Solutions, Lupton, AZ 86508, USA; janene.y@sixth-world.com; 8 Department of Environmental Science, University of Arizona, Tucson, AZ 85721, USA; kchief@arizona.edu

**Keywords:** qualitative research, culturally anchored, Navajo Nation, Indigenous, environmental disaster, community engaged research, decolonized research

## Abstract

The Gold King Mine Spill (Spill) occurred in August 2015 upstream from Silverton, Colorado and released three million gallons of contaminated water into the Animas River, a tributary to the San Juan River that flows across the Navajo Nation. Using principles of community-engaged research, the Gold King Mine Spill Diné Exposure Project co-developed a culturally anchored approach to conduct focus groups and analyze narratives collected in three Diné (Navajo) communities along the San Juan River within 9 months of the Spill. Focus group questions were designed to document the socio-cultural impacts of the Spill. This paper: (1) outlines the partnerships and approvals; (2) describes focus group design, training, data collection and analysis; and (3) reflects on the use of a culturally anchored approach in Indigenous, specifically Diné-centered research. Diné social and cultural etiquette and concepts of relationality were used to adapt standard (non-Indigenous) qualitative methods. Findings describe community perceptions of short-term impacts of the disaster, as well as past and present injustices, communication related to the Spill, and concerns of persistent threats to Diné lifeways. The culturally anchored approach was critical in fostering trust with Diné participants and aligned with the candor of the discussions.

## 1. Introduction

The Gold King Mine Spill Diné Exposure Project (GKMS-DEP), a collaboration among the University of Arizona, Northern Arizona University, Navajo Nation Community Health Representatives (CHRs), Diné College, Fort Lewis College, Tó Bee Nihi Dziil (a grassroots coalition) and the Navajo Nation navigated their partnership and research within a context of distrust grounded in past socio-cultural trauma and environmental injustices. Historically, research in Indigenous communities has negative connotations and for community members and leaders conjures up ethic violations and cultural insensitivity. Only recently has the scientific community been challenged to decolonize theoretical frameworks [1,2,3]. Decolonization is the process of dismantling and deconstructing colonial ideologies of the superiority of western ideals and methods by valuing Indigenous knowledge and approaches, eliminating western biases and assumptions and examining the researchers’ positionality with the Indigenous community [4]. Indigenous scholars have described approaches to data collection, analysis and interpretation informed by the cultural perspectives of the community involved in the research [2,3,5]. 

Scholars have been transparent in describing the tension between Indigenous and non-Indigenous ways of knowing [1,3,6,7,8]. Accordingly, suggestions for moving forward have been diverse. Some Indigenous scholars have outlined the ontological and epistemological aspects of Indigenous ways of knowing, actively challenging the colonial oppression of research grounded in western ideology and advocating for the sole or predominant use of Indigenous methods in research with Indigenous communities [1,8,9]. Others, often teams of Indigenous and non-Indigenous scholars, have navigated a framework described by Martin [2], as Two-Eyed Seeing, that embraces the contribution of Indigenous and non-Indigenous systems of inquiry. Mohatt et al. [7] defined their approach to grounding the research methodology in the culture and community, as culturally anchored research. Yet, Mohatt et al. [7] and Simonds and Christopher [3] raised concerns about the receptivity of grant reviewers to proposals that rely predominantly on Indigenous worldviews and frameworks of inquiry and interpretation. Although Indigenous methods of data collection, such as talking circles and storytelling, have gained acceptance in federally funded research, investigators often feel the need to include non-Indigenous methods and positivistic, quantitative design in their research plan to appeal to funding agencies [3,7,10,11].

The GKMS-DEP committed to maintaining the cultural context of participants’ statements. Thus, the Indigenous and non-Indigenous scholars involved in the project readily adopted Martin’s Two-Eyed Seeing approach, modifying standard western qualitative methods to incorporate Diné (Navajo) worldview and culturally guided etiquette for social interaction. Concurrently, applying Mohatt et al.’s [7] culturally anchored strategy assured that methodological decisions drew on Diné epistemology. This decolonized framework led by Diné cultural experts, traditionalists and medicine people, some of whom were also skilled researchers, guided the research approach for the GKMS-DEP. This paper: (1) outlines the partnerships and approvals needed to implement the project; (2) describes the culturally anchored approach designed for focus group design, training, data collection and analysis; and (3) reflects on the use of a culturally anchored approach in Indigenous-centered research.

## 2. Background

The university–tribal partnership designed GKMS-DEP to document environmental health and socio-cultural impacts on Diné communities from the release of three million gallons of acid mine drainage on 5 August 2015, from the Gold King Mine (located upstream from Silverton, CO, USA) into the Animas River, a tributary of the San Juan River that flows across the northern border of the Navajo Nation [12]. These rivers are important to Diné people, many of whom depend on the water for irrigated farming and raising livestock for food and income. More importantly, Diné people have a strong cultural and spiritual connection to the San Juan River that represents the male river on the Navajo Nation. [13] Central to the Diné connection with people and place is the principle Diné teaching of K’éí, which refers to descent, clanship and kinship, and involves exercising respect for all life forms on earth and in the cosmos [14]. A core element of this K’éí teaching is K’é, the honoring of individual and familial relationships to people and living things that guides the behaviors and interactions of Diné people with relatives [13,15,16]. With the guidance of Diné cultural experts who served as mentors for the researchers, these Diné philosophies were the foundation of efforts to develop a culturally anchored qualitative approach to conduct focus groups and analyze narratives from Diné communities after the Gold King Mine Spill (GKMS or Spill) of 2015. The outcome was intended to inform emergency planning, communication, and steps for community healing. 

The Navajo Nation, a term used to refer to both the collective people and the reservation, was recently acknowledged as the largest federally recognized Native American nation in the United States, with 399,494 enrolled Diné citizens [17]. The federally recognized name is “The Navajo Nation” [18] but the traditional name is Diné, which means “The People.” The Navajo Nation has the largest land base of any federally recognized Native American nation, located on over 27,000 square miles spanning the four corners area of Arizona, New Mexico, Colorado and Utah [19]. The Navajo Nation is a sovereign government comprising three branches: executive, legislative, and judicial. The executive branch is led by the Navajo Nation President and Vice President. The legislative branch is led by council delegates representing the 110 communities or local governmental chapters. The judicial branch consists of judicial courts using Diné traditional laws. Within the Navajo Nation, a chapter is the local form of government and is semi-autonomous, able to make decisions which concern their own chapter and its residents.

Coincidentally, only two days after the Spill occurred while briefing the Navajo Nation President Honorable Russell Begaye on the University of Arizona (UArizona) Superfund Research Program (SRP) community engagement and outreach on legacy mining on the Navajo Nation, the Navajo Nation Environmental Protection Agency (NNEPA) asked Karletta Chief (Diné), (Principal Investigator of the SRP Community Engagement Core) what would be the impact of the Spill on the Navajo people. Within days, Chief began attending the Navajo community public forums where Diné leaders, farmers and ranchers voiced their concerns. One of the main complaints was the lack of access to materials and data about the Spill and ineffective communication with the Diné people. In response, Chief worked with the UArizona Indian Cooperative Extension (ICE) Agent, based in Shiprock, NM, Navajo Nation to respond to most frequently asked questions from Diné farmers and wrote a fact sheet for Diné communities entitled Understanding the Gold King Mine Spill [20]. This fact sheet was distributed to Diné farmers by the ICE agent within two weeks of the Spill. Subsequently, the UArizona SRP offered research assistance to the Navajo Nation EPA and Navajo Water Department of Water Resources to address impacts of the Spill on the Navajo Nation. In response, the Navajo Nation Department of Water Resources requested concept papers from the UArizona on potential research topics. SRP submitted two concept papers identifying rapid federal research funding mechanisms. The first focused on evaluating the impacts of the GKMS on social, community and individual health of Diné peoples living along the Animas and San Juan rivers using a Diné worldview. The second concept paper evaluated the immediate and long-term GKMS impacts by investigating the fate and transport of heavy metals and arsenic from the Animas River (initial Spill entry point), along the San Juan River to final catchment at Lake Powell Dam. The Navajo Nation EPA selected the first concept paper and initiated the formation of a university–tribal research partnership that included the Navajo Nation CHR Program, Tó Bee Nihi Dziil, Diné College, Fort Lewis College, and Northern Arizona University to develop a research plan. The Navajo partners were involved in GKMS emergency response and community forums and hence provided valuable input in the proposals that addressed Diné community concerns to answer community questions. In addition, GKMS-DEP held listening sessions to seek input from impacted Diné communities [21]. 

Within two months of the Spill, the research concept to document both levels of contaminant exposure and perceptions of health risks, was presented and approved by the Shiprock Chapter, San Juan River Farm Board, San Juan River District 13 grazing committee, Nenahazaad Chapter, and the Northern Navajo Council with letters of support from the Navajo Nation President and Vice President, division directors, and community partners. This rapid approval reflected the community support for the research. Two initial grant proposals were developed by the multi-institutional team and submitted by the University of Arizona. Within 8 months of the Spill, the GKMS-DEP was funded by the National Institutes of Health’s National Institute of Environmental Health Sciences (NIH-NIEHS) time-sensitive R21 mechanism, and the University of Arizona Agnese Nelms Haury Program in Environment and Social Justice. With these resources, the GKMS-DEP could collect data to determine the levels of exposures to contaminants in three Navajo chapters downstream of the Spill, assess temporal and spatial changes in sediment, agricultural soil, river and well water in the same three Navajo chapters within 12 months of the Spill, [22] and determine the association between Navajo chapters members’ perception of health risks and measured health risks from the Spill [23,24,25]. 

Guided by the underlying principle of K’é, the GKMS-DEP’s goals were to empower Diné individuals with scientific knowledge, expand the diversity of voices responding to the Spill (e.g., community leaders as well as farmers, ranchers and non-land users), increase individual and community resilience by understanding and minimizing contaminate exposure, and contribute to tribal capacity by training Diné College students, environmental interns, and Community Health Representatives (CHRs) in quantitative and qualitative data collection. This interconnected framework, grounded in K’é, applies the concepts of mutual respect and working together as a family to identify solutions. With K’é in mind, the GKMS-DEP worked closely with Navajo chapters using a community-engaged framework, SNBH (SNBH is the acronym for a sacred Diné phrase. Respectfully, the authors have decided to use only the acronym), based on Diné fundamental teachings and life principles. SNBH, a “Beauty Way” phrase used in beauty-way ceremonies’ prayers, songs and teachings, is uttered for aspiration and goodwill for all [13]. SNBH has been referenced as the foundational basis of Diné-centered research methodologies such as the Hozhoogo Na’adah model, developed by Diné scholar and Diné College faculty Herbert J. Benally in 2008, and has been used to assess Navajo wellness projects and policies [26,27,28]. The use of SNBH is relevant and appropriate if efforts are for the betterment of humankind and Mother Nature, Diné are leading in the efforts and Diné are involved. SNBH-derived frameworks are guided by four interrelated and interdependent areas of knowledge (spiritual, work, family, home/environment) that align with four parts of the day. Health and wellness are found in the balance of these four areas. An SNBH epistemological lens enables a culturally based systematic examination of social and political issues impacting Navajo people and livelihoods for the purpose of fostering or restoring balance (health and wellness) [26].

Working with cultural experts, the qualitative data collection designed to understand the impacts of GKMS on Diné people was guided by relational principles key to the Diné worldview. Analysis was designed to interpret and maintain the context of the narratives and was informed by structuring scientifically accepted methods within a cultural framework. In this project, a team of Diné and non-Diné scholars worked collaboratively with Diné cultural experts to co-develop a research approach that centers Diné social and cultural protocols and Diné concepts of relationality to shape and anchor standard (non-Indigenous) methods of data collection and analysis. 

## 3. Methods

### 3.1. Question Development

In December 2015, general focus group questions addressing the impact of the GKMS on Diné people were framed based on concerns received from the UArizona ICE extension agent during Navajo farming meetings, conversations with Navajo leaders and community partners (particularly Navajo Nation CHRs and Tó Bee Nihi Dziil), audio from a Navajo Nation Council Session and a Navajo EPA meeting, and presentations to Navajo chapters, the Northern Navajo Agency Council, farming boards, and grazing districts. Academic researchers then framed the questions in a chronological framework: before the Spill, after the Spill and into the future. 

Given their previous conversations with victims of the Spill, Diné researchers and the Navajo Nation CHR Program advised the GKMS-DEP to document community members’ lifeways and relationship with the river before, during and after the Spill, and their post-Spill expectations. For example, what would you like to see for the San Juan River into the future? How do you see your community using the San Juan River in 50 years? Or 100 years? These questions were guided by three themes: Risk of Disruption, Change (Experience or Projected) and Future/Solutions. This approach allowed participants to speak to the risk of disruption to lifeways as caused by a loss of interest in farming due to forces of assimilation prior to the Spill or if disruption was initiated or accelerated by the Spill.

### 3.2. Institutional and Tribal Approval

This final set of questions was submitted to the Navajo Nation Human Research Review Board (NNHRRB) in January 2016 and approved in February 2016. NNHRRB, the University of Arizona Human Subjects Protection Program, and a ceded review agreement with Northern Arizona University approved all personnel, protocols, consents, recruitment materials, study instruments, and dissemination. 

### 3.3. Training

In February 2016, a focus group training was held in Flagstaff, AZ with 12 facilitators, note takers, recorders and assistants. Focus group team members were Diné and non-Diné undergraduate and graduate students, university faculty and staff and community members. The four facilitators were Diné and Diné Dinéke’ji yádałti’íígíí (fluent Diné language speakers). This session introduced standard, non-Indigenous approaches to conducting focus groups [29,30] and opened a discussion to develop additional guidelines to make focus group sessions culturally appropriate and to ensure that the cultural relevance of the conversations in Dinéke’ji was honored and maintained throughout the research process. To standardize the data collection process as much as possible, the four facilitators agreed on Diné words to use in the translation of focus group questions. 

By aligning with a Diné approach to problem solving, which emphasizes critical thinking and deep discussion around a challenge, developing a plan, implementing the plan, reflecting on the process, and changing it as needed [31,32,33], the group reached consensus and standardized a protocol for facilitators, note takers, recorders and assistants to ensure all sessions were comparable despite being conducted by different teams. Another training was held in May 2016 for note takers and assistants who were unable to attend the first training.

### 3.4. Recruitment

**Figure 1:**
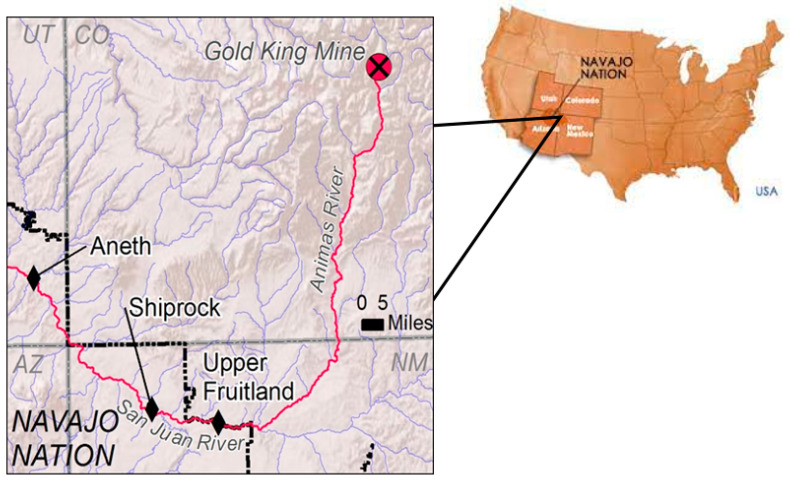
Map illustrating the location of Gold King Mine and Diné communities [24].

Twelve focus groups were conducted in the Diné communities or chapters of Upper Fruitland, NM, Shiprock, NM and Aneth, UT in May and June 2016. These chapters represent variations in immediacy of exposure to the Spill and response to the management of San Juan River irrigation water. Upper Fruitland, NM, located on the northeast side of the Navajo Nation at the point where the San Juan River enters the Nation, voted to re-open the irrigation canals after the Spill. Shiprock, NM, located 20 miles downstream from Upper Fruitland, voted to keep irrigation canals closed and allowed their field to fallow, as their canals were closed longer than other communities along the river. Aneth, UT, located on the west side of the Nation where the river exits the Navajo Nation, has community wells that could be used for local water needs. See Figure 1. 

Dates of the focus groups were established in collaboration with chapter house officials as community buildings were used for the focus groups. Flyers and newspaper and radio announcements explained the purpose of the focus groups, and provided the dates, times and inclusion criteria; specifically, participants had to be >18 years of age and reside in one of the three communities. Recruitment flyers written in English were posted in the three communities, information was broadcast on the Navajo Nation radio station (KTNN) in English and Dinéke’ji, and notices were placed in the Navajo Times. Participants were compensated with a $25 gift card. 

### 3.5. Data Collection 

Permission was requested from the chapter house officials for the use of local chapter houses during times when community members could attend, generally evenings and weekends. Most chapter house members live within less than 20 miles of their respective chapter house, thus minimizing the need for long travel times and mileage costs. In most cases, participants had been in the chapter houses on prior occasions for chapter meetings so the setting was familiar.

All sessions were implemented by teams consisting of both Diné and non-Diné team members. Per Diné cultural protocol, coffee, juice, water and in some cases, food, were provided throughout the sessions. All potential participants were greeted with a handshake, the traditional Diné greeting for all genders and ages, and verbal appreciation for coming to the sessions. Once potential participants were seated around a table, the facilitator, fluent in Dinéke’ji, opened with ádeehooldilzin, a personal greeting that establishes K’éí by identifying their matrilineal and patrilineal clans, identifying their ancestral communities and recognizing kinship to the focus group participants. Diné note takers introduced themselves in the same way, while non-Diné members provided general introductions that followed an acknowledgement of their matrilineal and patrilineal lineages and their place of residence or hometown. The formal introduction established K’é, which informs people of their relationality within Diné society and how they should interact through kinship [34]. 

The facilitator verbally explained that the focus groups were being conducted in response to community concerns and the compiled responses would be presented back to communities. Furthermore, each facilitator emphasized that the research was being led by Diné scholars in an effort to reduce the bias and inaccurate research often conducted in Diné communities. The facilitator reviewed the consent form in English and Dinéke’ji, including a clear statement that conversations were to be digitally recorded and handwritten by two note takers in the room (Diné and non-Diné). Potential participants were encouraged to ask clarifying questions and told they could leave without penalty if they did not wish to participate and have their statements recorded. The facilitator collected signed consent forms. 

Once the participants provided consent, the facilitator offered an opening prayer to acknowledge that all participants and facilitators were present to speak openly about the impact of the Spill and contribute to healing the Diné people. Then, moving clockwise around the room, acknowledging the Diné teaching that mountains songs and prayers were made to go clockwise, the path of the sun, from East to North, and back to the East [13], participants introduced themselves, stating their clans, name and often how long they had lived in the area (e.g., all their lives, left for a period of time for work or school, or married into the area). 

The focus group questions and prompts were posed to the group first in Dinéke’ji and then in English. Depending on their preference, participants responded in Dinéke’ji, English or a mix of both languages (code switching) [35]. Since all sessions were recorded, note takers were instructed to capture central statements of the speakers and to document non-verbal behaviors. Facilitators summarized Dinéke’ji to English and vice versa for all attendees, so monolingual speakers, whether Diné or non-Diné, would have some comprehension of the conversation. Initially, team members observed that some young adults did not talk or said little. They may not have felt comfortable talking in English when Dinéke’ji was primarily spoken in the session or when the groups had several elders. After this observation, the team, organized focus groups by Dinéke’ji fluency, with conversational Dinéke’ji speakers in one group and English-dominant speakers in the other. 

### 3.6. Data Processing

All digitally recorded sessions were transcribed verbatim, using the written notes to supplement when a recorded segment was not clear. A data quality (DQ) protocol was developed to correct and standardize spelling and terms, identify the context of places, and write out acronyms. When Dinéke’ji was spoken, transcribers indicated time and need for translator. Diné linguists were hired to translate Dinéke’ji statements into English. Guided by Diné connectivity and relationship to the environment, translation often required a phrase in English to convey concepts of pollution and loss, relying on Diné scholars’ previous work related to uranium pollution [36]. More than 14 h of recordings were transcribed, including 4.5 h in Dinéke’ji. Diné statements, once translated to English, were italicized. Each transcript was assigned to a member of the qualitative analysis team to apply the DQ protocol. Once completed, the transcript was placed in a Dropbox^®^ folder of DQ transcripts. 

### 3.7. Data Analysis

Transcribed and translated text were analyzed using NVivo^®^ 10 [37] and 11. [38] The nine-person qualitative analysis team was composed of six Diné (1 faculty and 5 graduate students) and three non-Diné (2 faculty and 1 graduate student) coders. Disciplines represented by the team members were hydrology, anthropology, environmental sociology, public health, environmental science, applied Indigenous studies and linguistics. The project-specific codebook was developed using a culturally informed, systematic, consensus-driven approach. Initially, using the three pre-designated themes of Risk, Change and Future/Solutions, all team members free-coded the same transcript. Each coder grouped phrases according to independently proposed sub-themes or child nodes. Collectively over several meetings, the coders identified commonalities of the independently derived sub-themes and discussed sub-themes that would best capture the concepts identified by each coder. 

**Figure 2:**
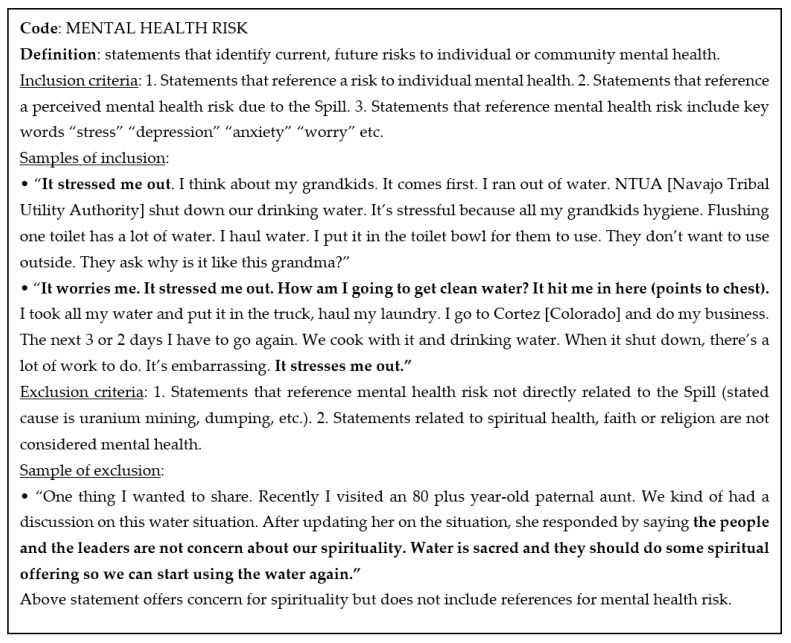
Excerpt from GKMS codebook developed for analysis of focus group narratives, Mental Health Risk.

**Figure 3:**
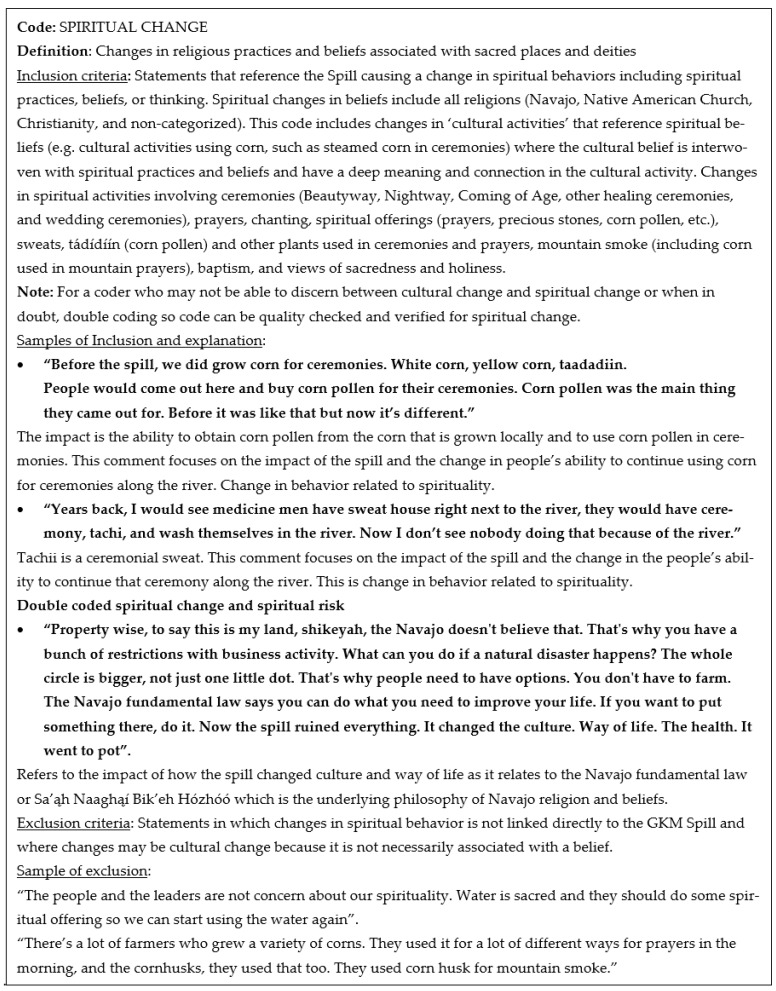
Excerpt from GKMS codebook developed for analysis of focus group narratives, Spiritual Change.

Once the team agreed on the initial set of sub-themes or codes, the codebook was developed. This process involved assigning two to three codes to each team member who would develop a draft definition and provide one to two examples of phrases that fit the code description and those that did not. In some cases, examples were provided of phrases that should be double coded. See Figure 2 and Figure 3. The team members collectively reviewed the draft code definitions and examples over a series of weekly meetings where criteria were refined iteratively based on discussion and consensus. Definitions and criteria would be clarified, expanded and in some cases, and new themes were developed and defined. In these discussions, non-Diné coders shared their challenges in distinguishing between phrases that referenced cultural or spiritual concepts. Non-Diné coders tended to double code these phrases, yet Diné coders generally agreed that some phrases were clearly cultural or spiritual and should not be double coded. This realization that non-Diné coders may lack the cultural background to make these distinctions, informed the analysis process. 

Pairs of coders, one Diné and one non-Diné, were assigned to code the same transcripts. The pairs were created without regard to field of expertise, university, previous coding experience or academic level (e.g., faculty or graduate student). The pairing process reflected the perspective of the team, respecting the insight of all coders and recognizing that previous coding experience and cultural insight were equally valuable to the validity of the outcomes. The pairs independently coded their assigned transcripts and then met in-person, via Zoom^®^, or on the phone to discuss their results and reach consensus on sub-theme coding, resulting in a single set of coding outcomes. If consensus could not be reached, the passages would be shared with the larger group at the weekly meetings and the group would reach consensus. 

## 4. Results 

### 4.1. Recruitment

The success of participant recruitment varied across the three communities. Shiprock Chapter residents were quite enthusiastic. For the first focus group in their community, more people attended than expected. The team had difficulty keeping the group to a manageable size (10–12) and had to request late arriving residents not to enter the group, which already had 22 participants. In addition, some residents requested to attend a second time, returning to later focus groups with friends and family stating they wanted to listen to others concerns. This request, which was accommodated if they did not contribute a second time, indicated a deep need for community members to state their concerns and hear about collective and unique concerns. However, at Upper Fruitland, NM, recruitment was less successful and at Aneth, UT, recruitment was difficult. As the teams arrived in the Upper Fruitland and Aneth Chapters, frequently no potential participants were present. Often team members who spoke Dinéke’ji went to local gas stations, convenience stores, and senior centers to distribute the flyers and verbally recruit participants. Anecdotally, older Dinéke’ji-speaking project personnel appeared to be more successful in on-site recruitment than Diné students, who were less fluent Dinéke’ji speakers. This observation aligns with cultural teachings and the enhanced credibility afforded to Dinéke’ji speakers. This on-the-scene approach was successful, yielding groups of 8–15 individuals for each focus group. 

### 4.2. Data Collection

Focus group length varied from 90 to 200 min. A hundred and twenty-three (123) Diné adults participated in 12 focus groups: four groups in Upper Fruitland, NM, six groups in Shiprock, NM and two groups in Aneth, UT. Table 1 provides descriptive information on age and land use status of participants. 

### 4.3. Data Analysis

Inter-coder reliability or the degree of agreement between coders was high. The percent of agreement in the number of units of agreement divided by the total units of measure within the data item or code, ranged from 74.41% to 100%, with a mean of 92.68%. Due to the difference in participant numbers at each site and the relatively small number of participants at Aneth, the analysis aggregated the responses of the participants. 

Guided by the three pre-designated themes of Risk of Disruption, Change and Future/Solutions, analysts identified 19 secondary or sub-themes. The secondary themes are listed in rank order in Table 2. Within the secondary theme of Cultural Risk of Disruption, tertiary themes were identified. The analysis team determined that a fourth primary theme, Distrust, emerged in the narratives. The statements could not be assigned to the pre-designated primary themes of Risk of Disruption, Change and Future, necessitating the development of a fourth theme: Distrust. 

**Figure 4:**
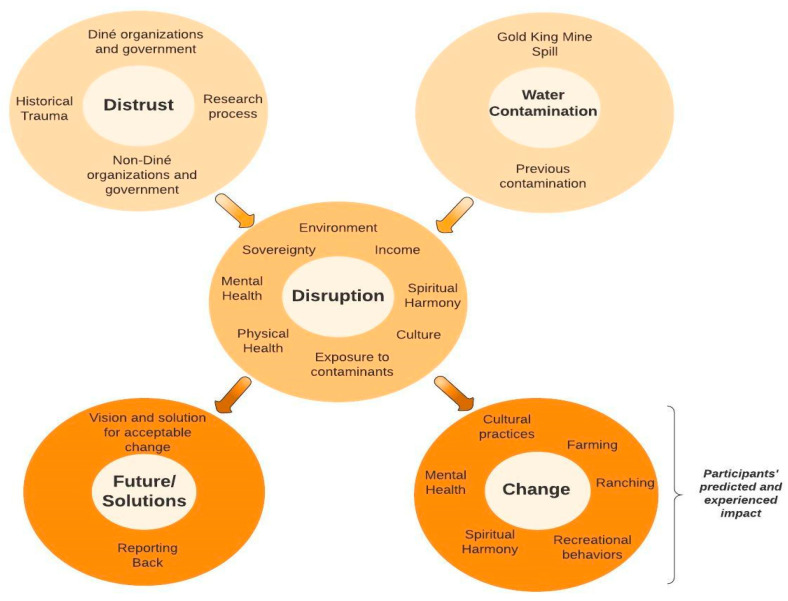
Combined impact of the Gold King Mine Spill water contamination and distrust contributing to risk of disruption and ensuing change and solutions.

The intention of this initial analysis process was to conceptualize the connectedness of these themes or concepts to inform the process of community healing and to support future developments in health risk communication within the Navajo Nation. The process began with the Diné Hataalii Association (DHA) a non-profit organization that exists to protect, preserve and promote Diné culture and spiritual practices for current and future generations. The DHA consists of over 200 Diné spiritual leaders and traditional practitioners. In presenting preliminary results to DHA in April 2019, DHA provided critical feedback in the need to center the results in Diné traditional worldview and using SBNH. Drawing on Diné worldview and the reciprocal relationship with the environment, as well as Billot et al.’s [39] work on the impact of Indigenous loss of connection to the land, Figure 4 is proposed as a visual depiction of the factors leading to risk of disruption and the ensuing outcomes. 

Interconnectedness: As depicted in Figure 4, adverse forces fueled the risk of disruption, specifically water contamination as well as on-going and heighted distrust of Diné and non-Diné entities and researchers, rooted in past historical injustices. Disruption was discussed in relationship to culture, the environment, exposure to contaminants, finances and income, mental health, physical health, sovereignty, and spiritual harmony. 

Participants predicted and experienced the impact of the disruption, which was change. Change was discussed in relationship to cultural practices, farming and ranching, mental health, recreational behaviors, and spiritual harmony. 

No change: As indicated, some participants relayed that Diné people had weathered similar assaults to their environment and culture, and change was inevitable, prompted by the Spill or not. Although this view was infrequent, the perspective is noteworthy and speaks to the pervasive notion of resilience and survival.

## 5. Discussion

### 5.1. Culturally Anchored Research Environment

The project team discussed and made purposeful decisions to create an inviting social and physical environment, using familiar spaces and cultural protocols. Honoring Diné relationship-centered practice, the team discussed and developed written protocols to ensure all sessions provided an opportunity for each participant, facilitator and support personnel to provide traditional introductions, so participants knew if they were related to fellow participants or project team members through direct family lineage or clan relationships. Holding sessions in a circle is common in focus group implementation but ensuring introductions proceeded in a clockwise fashion, again signaled to participants that the project protocols were guided by Diné etiquette. 

Ensuring that all facilitators were bilingual and able to translate the content of the discussion in real time, were able to demonstrate to participants that they could speak in Dinéke’ji, English or both and their statements would be understood and weighted equitably. 

Diné leadership and guidance of the project was demonstrated by the facilitators’ verbal review of the consent form and highlighting that one of the principal investigators was a Diné scientist, well known in the Nation. Furthermore, facilitators stated that many of the project investigators and most of support staff were Diné. The effort to create a Diné-centric environment may have encouraged the considerable discussion of distrust. Distrust was expressed in relation to Diné and non-Diné leadership, to past local and Navajo Nation-wide injustices, and even the research process that involved these focus groups and the water and soil sampling. As noted by tertiary themes, culture and distrust were discussed in depth and required further delineation.

The value of this approach is best illustrated by continued discussion with stakeholders, specifically Navajo Nation-elected and appointed leadership, traditional leaders and grassroots leadership. The GKMS-DEP team has provided results of the environmental, socio-cultural and risk assessment research in executive sessions. In these meetings, stakeholders agreed that communities need to be at the center of the emergency response and not be treated as though they do not understand the data and the science. As the Navajo Nation moves forward, continued Diné leadership in research and policy development was described as key to fostering community capacity and agency to address disasters and to averting widespread uncertainty and fear. 

### 5.2. Breadth of Analytical Perspective

The culturally anchored and multi-disciplinary approach to the analysis process of the qualitative data yielded a codebook that considered a breadth of concerns within the themes of risk of disruption, change and future, and allowed the theme of distrust to emerge. The diverse disciplines represented in the analysis team yielded all themes and in particular, risk of disruption as being interpreted and equitably valued as a cultural, financial, environmental, and physical experience.

The engagement of Diné and non-Diné investigators and students supported the integration of emic and etic insights as well as perspectives of experienced and emerging scholars into the development of the codebook and interpretation of narratives. Emic refers to an insider’s interpretation or perspective while etic refers to an outsider’s interpretation of an event or observation [40]. 

### 5.3. Disruption and Distrust

The prominence of risk of disruption to culture and change as well as distrust, reflects participants’ focus on the totality of the contaminated water’s impact. Although household and financial harm was discussed in relation to lost food, income and additional expenses required for moving livestock and hauling water, most participants expressed concern about the long-term impact on the culture. They posed and grappled with questions that expressed concern about the impact: How will the contamination impact the Diné people’s relationship with the rivers? Will the Diné people be able to continue their traditional subsistence patterns of farms and ranching? Will their children move away as they cannot continue the traditions? Will they become disconnected from family, the community, and traditions? 

The theme of disruption aligns with Kirsch’s [41] concept of culture loss. Kirsch [41] speaks of Indigenous culture loss as akin to intimate loss in relation to kinship and belonging rather than possession. Culture encompasses lifeways, subsistence practices, systems of meaning, social dynamics and identity, and cannot be separated from its geographic location [42]. The prominence and sub-themes of Risk of Disruption to Culture reflect the participants’ concern with the comprehensive scope and driving impact of the Spill. Reports developed by non-Diné correspondents often highlight the damage to crops and the costs related to livestock management, both which are grave burdens for the Dine people. Yet, the culturally anchored approach used in the GKMS Diné Exposure Project data collection, analysis and interpretation no doubt contributed to participants’ comfort with sharing the greater burden of long-standing, unresolved disruptions and distress. 

### 5.4. Broader Implications for Community Collaboration

In response to an environmental disaster, this project built trust between a Diné and non- Diné team and Diné communities by allowing the methodological modifications and interpretations to be guided by Diné fundamental teachings and life principles. Honoring Diné ways of knowing communicated the research team’s respect for the Diné people and culture and supported an open, transparent exchange of concerns and information. This relationship has led to future collaborative projects that also address community concerns, as related to food sovereignty and resilience during disasters and pandemics.

## 6. Conclusions

The GKMS Diné Exposure Project developed a culturally anchored and multi-disciplinary approach to the planning, collection and analysis of the narratives shared by the Diné citizens living in select communities along the San Juan River contaminated by the 2015 Gold King Mine Spill. The team used a Two-Eyed Seeing [2] approach that embraced the contribution of Indigenous and non-Indigenous systems of inquiry. In addition, the attention to Diné social and cultural etiquette created a comfortable, safe space for participants to openly discuss past and present injustices, opinions of Diné and non-Diné leadership response and communication related to the spill, and concerns of the persistent threats to Diné culture and lifeways. The conversations did not stay focused on the Spill and water contamination but transcended the incident to reignite and evoke stories of other environmental contaminations, relocation, broken treaties and lack of agency and voice, adding to a long legacy of inter-generational trauma. Essentially, the participants encouraged the project to document and examine the Spill not as a single incident but as a recent example in a long history of assaults. 

Grounded in standard qualitative research methods but organic in the inclusion of Diné relationships, protocols, bilingual and bicultural competencies, and worldview, this culturally anchored, co-designed research approach reflected the strength and the commitment of the Diné and non-Diné team members to be guided by Diné cultural experts and to honor the diversity of disciplines and cultures, yielding culturally truthful (sound) and scientifically valid outcomes [43]. Similarly, the approach created a means for the participants to broaden the initial emphasis of the focus groups and to describe the socio-cultural impact of the Spill within a continuum and within the context of the socio-political environment.

## Figures and Tables

**Table 1:** Age and Land Use Status of Focus Group Participant by Chapter.

Chapter	Total N	Young Adults (18–34 Years)	Adults (35–65 Years)	Elders (>65 Years)	Land Users (Farmers, Ranchers and Gardeners)
Upper Fruitland, NM	39	13%	30%	57%	56%
Shiprock, NM	67	20%	24%	56%	12%
Aneth, UT	18	21%	42%	37%	78%

**Table 2:** Themes and sub-themes derived from analysis of 12 focus groups held in Shiprock, NM, Upper Fruitland, NM and Aneth, UT in May and June 2016.

Risk of Disruption	Projected Change	Future	Distrust
Culture Community Family Identity River	Culture	Vision for acceptable change	Historical trauma Diné organizations and government Non-Diné organizations and government Research process
Environment	Farming	Solution for acceptable change	
Exposure to contaminants	Mental Health	Reporting back	
Finances and income	Ranching		
Mental health	Recreational practices		
Physical health	Spiritual harmony		
Sovereignty	No Change		
Spiritual harmony			
No Risk			

## Publication Approval

The Navajo Nation Human Research Review Board approved the publication of this manuscript on 19 January 2021.
